# The emerging role of glycolysis and immune evasion in ovarian cancer

**DOI:** 10.1186/s12935-025-03698-x

**Published:** 2025-03-05

**Authors:** Bowen Jin, Zehua Miao, Junjie Pan, Zhen Zhang, Yibei Yang, Yidong Zhou, Yuanxiang Jin, Zheng Niu, Qiaoping Xu

**Affiliations:** 1 Department of Clinical Pharmacology, Key Laboratory of Clinical Cancer Pharmacology and Toxicology Research of Zhejiang Province, Affiliated Hangzhou First People’s Hospital, Cancer Center, Westlake University School of Medicine, Hangzhou, 310006 China; 2 Fourth Clinical Medical College of Zhejiang Chinese Medical University, Hangzhou, China; 3 Dalian Medical University, Dalian, China; 4 Department of Oncology, Hangzhou Cancer Hospital, Zhejiang, Hangzhou, 310002 China; 5 Department of Gynecology, Affiliated Hangzhou First People’s Hospital, Cancer Center, Westlake University School of Medicine, Hangzhou, 310006 China; 6 Department of Clinical Pharmacology, Key Laboratory of Clinical Cancer Pharmacology and Toxicology Research of Zhejiang Province, Affiliated Hangzhou First People’s Hospital, Westlake University School of Medicine, Hangzhou, China

**Keywords:** Ovarian cancer, Glycolysis, Microenvironment, Immune evasion, Immunotherapy

## Abstract

Ovarian cancer (OC) is one of the three most common malignant tumors of the female reproductive system, with the highest mortality rate among gynecologic malignancies. Like other tumors, OC cells undergo metabolic reprogramming phenomenon and convert glucose metabolism into “aerobic glycolysis” and generate a high concentration of lactate, i.e., the “Warburg effect”, which provides a large amount of energy and corresponding intermediary metabolites for their survival, reproduction and metastasis. Numerous studies have shown that targeted inhibition of aerobic glycolysis and lactate metabolism is a promising strategy to enhance the sensitivity of cancer cells to immunotherapy. Therefore, this review summarizes the metabolic features of glycolysis in OC cells and highlights how abnormal lactate concentration affects the differentiation, metabolism, and function of infiltrating immune cells, which contributes to immunosuppression, and how targeted inhibition of this phenomenon may be a potential strategy to enhance the therapeutic efficacy of OC.

## Introduction

The metabolic activities of malignant tumor cells differ significantly from those of normal cells, and this special metabolic pattern is known as the phenomenon of tumor metabolic reprogramming [1]. Glucose as the most important material energy source of the cell, in normal cells is mainly through the oxidative phosphorylation metabolic pathway to consume glucose to produce energy, but in malignant tumor cells even in the oxygen-supplied sufficient environment, will be through a series of glycolysis under the action of glucose consumption of large quantities of glucose and the production of large quantities of lactate, so as to rapidly obtain energy, this metabolism is called aerobic glycolysis, namely “Warburg effect”, which is one of the first metabolic reprogramming discovered [2, 3]. Furthermore, this aerobic glycolysis can provide a large amount of energy as well as corresponding intermediate metabolites for metabolic processes such as tumor cell survival and reproduction [4, 5]. The end product of aerobic glycolysis is lactate, which acidifies the tumor microenvironment (TME) and can act as a multifunctional small molecule chemical to promote tumor development [6].

Ovarian cancer (OC) is a malignant neoplastic disease that occurs in the ovaries and is the most common malignant tumor in women, which is a major challenge that threatens the life and health of women [7]. Despite the development of early diagnosis and treatment methods, the overall incidence is still rising, and the five-year survival rate in Asian countries remains low at 59.6% [8]. OC has a unique TME, which is closely related to tumor proliferation, invasion and metastasis. A study showed that numerous cytokines in follicular fluid such as insulin-like growth factors (IGF), epidermal growth factor (EGF) and transforming growth factor-β (TGF-β) induce tumor cell proliferation and invasion [9]. Furthermore, OC has a high rate of peritoneal metastasis, and the fatty acid-rich peritoneal microenvironment composed of peritoneal fluid and peritoneum creates favorable conditions for the progression and metastasis of OC [10, 11], and alterations in glycolytic processes also create a facility for the development of peritoneal metastasis of OC, which distinguishes it from other modes of cancer metastasis [12]. Most OC originates from ovarian epithelial cells, and like other tumors, it exhibits the “Warburg effect”. OC cells create a localized hypoxic, hypoglycemic and acidified environment by consuming large amounts of glucose through aerobic glycolysis and generating the end product lactate. The acidified TME inhibits the activity of immune cells and helps OC cells evade the immune system, promoting their metastasis and invasion [11]. Further understanding of the effects of glycolysis and its metabolites on immune cell function in OC could provide better ideas for rational immunotherapy, leading to better efficacy.

## Characteristic of aerobic glycolysis in OC

### Enhancement of aerobic glycolysis in OC

Cancer cells follow the “Warburg effect”, and their metabolism is significantly different from that of normal cells. Fong et al. [13] used gas chromatography/mass spectrometry (GC/MS) and liquid chromatography tandem mass spectrometry (LC/MS/MS) methods to report the difference of metabolites between normal ovary and OC. Several intermediate metabolites and their derivatives in aerobic glycolysis, such as erythronate, fumarate, malate, etc. were found to be differential metabolites, which were significantly increased in cancer tissues. The altered glucose metabolism may serve as a crucial factor for distinguishing OC cells from normal cells. In another study, Cheng et al. [14] used LC-MS on serum from patients with OC and benign ovarian tumors and found that altered metabolites such as maltose, maltotriose, raffinose, and mannitol, which further caused disruption of the tricarboxylic acid (TCA) cycle, could differentiate benign tumors. Similarly, other researchers have observed abnormalities in glucose metabolism in OC [15, 16]. Loar P et al. [17] demonstrated that inhibition of glycolysis in OC cells enhances the effect of chemotherapeutic agents acting on cancer cells to cause apoptosis, and that the glycolytic phenotype of OC cells may be associated with chemotherapy resistance and could be targeted for therapeutic intervention. Wu et al. [18] further confirmed that Paris saponins VII (PSVII) inhibited glycolysis in OC cells, thus inhibiting cancer cell proliferation, inducing apoptosis and exerting its anticancer effect.

Proto-oncogenes and tumor suppressor genes can regulate cellular glucose metabolism. The development of cancer is closely related to the activation of proto-oncogenes and the inactivation of tumor suppressor genes, which cause corresponding changes in cellular glucose metabolism. Activation of the proto-oncogene c-MYC activates cellular glycolysis by transcriptase-upregulating the expression of key glycolytic enzymes, including glucose transporter 1(GLUT1), lactate dehydrogenase A (LDHA), and some key points of glucose metabolism such as hexokinase 2 (HK2) and pyruvate dehydrogenase kinase 1(PDK1) [19]. Human pituitary tumor transforming gene (PTTG), as a proto-oncogene, is involved in the occurrence, invasion and metastasis of OC. Wang et al. [20] found that the expression level of PTTG was synchronized with aerobic glycolysis in OC cells, and the expression of c-MYC and several key enzymes of aerobic glycolysis, such as GLUT1, LDHA and pyruvate Kinase M2 (PKM2), was down-regulated with the inhibition of PTTG, and oxidative phosphorylation was increased. Han et al. [21] showed that p53 regulates the intracellular transport of HK2, and in chemo-sensitive OC cells, phosphorylated p53 promotes the translocation of HK2 and apoptosis-inducing factor (AIF) from the mitochondria to the nucleus, which inhibits HK2-mediated glucose metabolism and induces apoptosis in cancer cells. The TP53 gene up-regulated TIGAR while down-regulating the expression of GLUT1 and some key enzymes of glycolysis, thereby reducing glucose consumption and lactate production in OC cells and inhibiting glycolysis [22]. Deletion of the tumor suppressor gene BRCA1 increases glycolytic activity by inducing the transcription factors MYC and STAT3 to activate HK2 expression [23]. In addition, studies have found that PI3K/AKT signaling activation plays an important role in tumor aerobic glycolysis, which can regulate the activity and expression of some glycolytic enzymes such as HK2, PFK, and PK. Oncogene ACTL6A not only mediates glucose utilization, lactate production and pyruvate levels by regulating PGK1, but also can be up-regulated by follicle-stimulating hormone through the PI3K/AKT pathway to promote glycolysis of OC [24]. In contrast to normal cells, cancer cells obtain their required energy mainly through glycolysis even under aerobic conditions. Although it is a much less efficient form of energy production than mitochondrial OXPHOS, the high level of glycolysis can provide intermediates and macromolecules for cancer cell biosynthesis, and these changes are considered to be the evolutionary advantages of cancer cells [25].

**Fig. 1:**
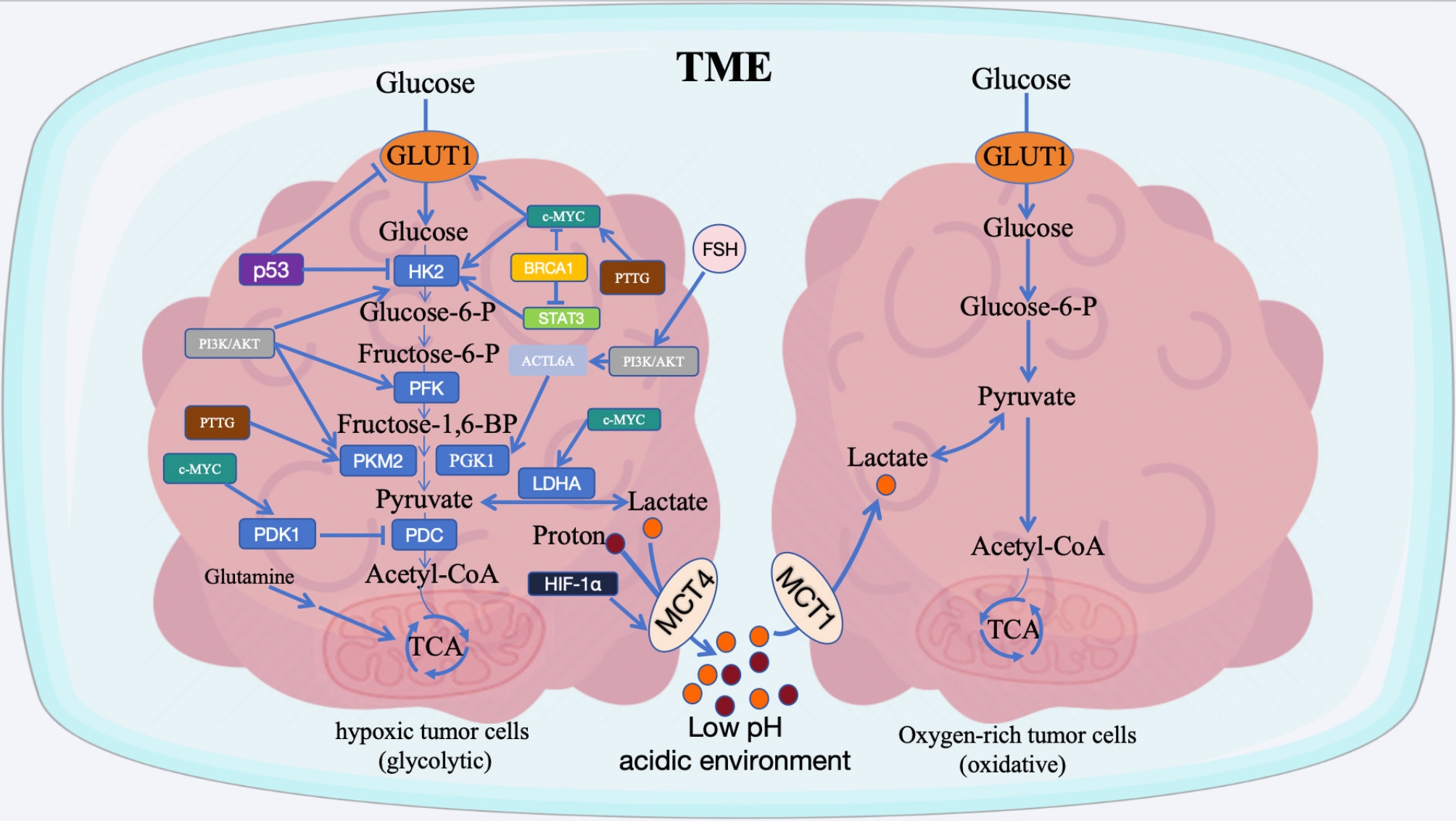
Regulation of lactate metabolism by oncogenes as well as other factors and the “lactate exchange” phenomenon between tumor cells. Hypoxic tumor cells (glycolytic type) can utilize glycolysis and glutamine as energy sources and transport large amounts of lactate produced to the TME through the high expression of MCT4, leaving it in a low pH acidic environment and inhibiting immune cell function and metabolism. Oxygen-rich tumor cells (oxidative type) preferred to take up lactate through high expression of MCT1 and gained energy through oxidation of lactate and TCA cycle

In addition, some studies have shown that cancer cells can alter TME to improve the fitness of cancer cells and their growth advantage through the lactate produced by glycolysis. Lactate derived from tumor cells can be released into the extracellular microenvironment in large quantities, resulting in a concentration of lactate 10 times that of the normal tissue microenvironment, and the pH value can be as low as 6.0-6.8 [26]. Battista et al. [27] detected that the lactate concentration of OC patients ranged from 17.51–37.16µmol/g, which was significantly higher than that of benign samples (5.43–6.54µmol/g). Due to the massive consumption of nutrients by tumor cells, when solid tumor tissues are still in the stage of insufficient vascular supply, they usually present a state of nutrient deficiency. Therefore, tumor cells require multiple mechanisms to maintain their own energy supply, including the phenomenon of “lactate exchange” between tumor cells in different tissue regions [28]. The TME shows high heterogeneity. According to the different oxygen levels in the extracellular environment, tumor cells can be divided into oxygen-rich tumor cells (oxidative type) or hypoxic tumor cells (glycolytic type). For both glycolytic and oxidized cancer cells, the use of alternative molecules such as lactate or glutamine as an energy source depends on the anatomical distribution of tumor cells, with glycolytic cancer cells located further away from blood vessels, whereas oxidized cancer cells are located around blood vessels [29]. Monocarboxylic transporters (MCTS) are crucial for transporting lactate and protons to the TME in cancer cells [30]. Specifically, tumor cells located deep in the TME, far from the oxygen supply in the blood, can utilize glycolysis and glutamine as energy sources and transport large amount of lactate to the extracellular environment by high expression of MCT4, while cancer cells located close to blood vessels with normal oxygen supply are more likely to take up lactate by high expression of MCT1 and energy is obtained through the oxidation of lactate in mitochondria and the TCA cycle [31–33]. Hypoxic cancer cells with a glycolytic phenotype promote the high expression of MCT4 by up-regulating hypoxia-inducible factor-1α (HIF-1α), which leads to rapid lactate transport [34]. Furthermore, inhibition of MCT1 impaired tumor-cell growth and glycolysis, reduced glucose transport, and decreased energy production and glutathione (GSH) levels. The intracellular hydrogen peroxide level increases with the decrease of GSH, resulting in mitochondrial damage and cell death [35]. Thus, lactate transport through the TME between different cell populations plays a crucial role in tumor cell growth and progression (Fig. 1). Lactate, the metabolite of tumor cells, accumulates in the TME, which can inhibit the function and metabolism of immune cells and induce tumor immunosuppressive microenvironment [36]. A growing body of research suggests that lactate and the acidic environment it creates have a broad and important role in the regulation of various natural immune cells in tumor tissues.

### Key enzymes in aerobic glycolysis in OC

**Fig. 2:**
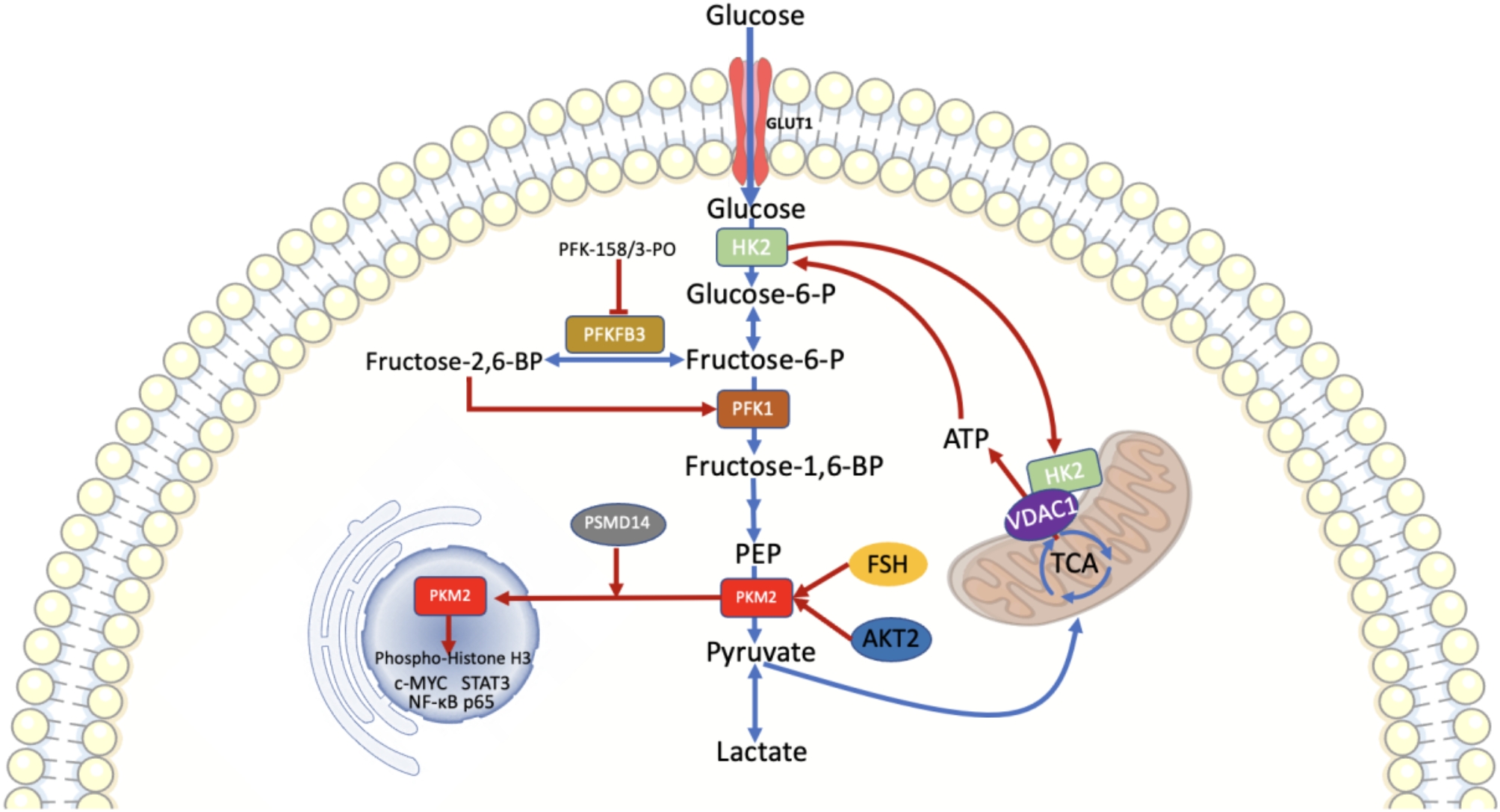
Three rate-limiting enzymes in aerobic glycolysis. HK2, PFK1, and PKM2 are the three rate-limiting enzymes involved in glycolysis. HK2 catalyzes the conversion of glucose to G-6-P and binds to VDAC1 on the outer mitochondrial membrane, which allows preferential entry of ATP into the HK2-mediated glycolytic step via the open VDAC1. PFK1 catalyzes the conversion of F-6-P to F-1,6-BP, and its activity is regulated by the catalytic product F-2,6-BP of PFKFB3. PKM2 not only catalyzes PEP to pyruvate, but also translocates to the nucleus and acts as a protein kinase to phosphorylate histone H3 and activate various gene transcripts, such as c-MYC, STAT3, and NF-κB, which indirectly supports aerobic glycolysis and promotes the progression of tumor cell proliferation

Aerobic glycolysis has three rate-limiting enzymes, hexokinase (HK), phosphofructokinase (PFK) and pyruvate kinases (PKs) (Fig. 2). Changes in the expression of these enzymes significantly influence the progression of OC.

### Hexokinase 2 (HK2)

HK2, the first key rate-limiting enzyme of the “Warburg effect”, catalyzes the conversion of glucose to G-6-P, which is highly expressed in OC tissues and correlates directly with advanced and high-grade cancers and plays a role as an independent prognostic factor [37]. HK2 can bind to the Voltage-Dependent Anion Channel 1 (VDAC1), which inhibits the movement of the VDAC1 N-terminal helix and permits ATP to preferentially enter the HK2-mediated glycolytic step via the open VDAC1, thereby promoting aerobic glycolysis [38]. HK2 expression in OC is regulated by a variety of signaling pathways and transcription factors, including the PI3K/AKT signaling pathway, GPNMB/HIF-1α axis, STAT3 and miR-603 [39–42].

In OC, elevated HK2 expression can lead to chemoresistance. Yu et al. [43] demonstrated that mutations in the UBA structural domain of the autophagy receptor p62 altered the overall abundance and mitochondrial localization of HK2 in cells, which increased the phosphorylated ubiquitin form of parkin, stabilized mitochondrial autophagic activity, and ultimately induced cisplatin resistance. However, knockdown of HK2 could limit cisplatin-induced ERK1/2 phosphorylation and autophagy, which reduced cisplatin resistance in OC cells [44]. In addition, Li et al. [45] found that increased HK2 expression and binding to VDAC1 induced by the SBD structural domain of HSP70 maintained the integrity of the mitochondrial permeability transition pore (MPTP), which reduced apoptosis and promoted cisplatin resistance in OC. Based on its critical role in OC, HK2 is considered a promising metabolic target for the development of new OC therapeutic approaches.

### Phosphofructokinase-1 (PFK1)

Phosphofructokinase-1 (PFK1) serves as the second rate-limiting enzyme in glycolysis and exhibits the most potent regulatory influence on glycolysis metabolism, which catalyzes the conversion of Fructose-6-P into Fructose-1,6-BP. There are three PFK1 isoforms existing in mammals, including PFK-L (liver), PFK-M (muscle), and PFK-P (platelet), and the varying expression levels of these isoforms in human tissues are potentially associated with energy metabolism requirements [46].

PFK1 is active as a tetramer but less active as a monomer and dimer, and this structural transition in PFK1 largely influences glycolytic fluxes [47]. For example, downstream products of the PFK1 enzyme, including high concentrations of ATP, phosphoenolpyruvate (PEP), citrate, and lactate, can induce the dissociation of the PFK1 tetramer into a dimer, leading to the inhibition of PFK1 activity and providing negative feedback to the glycolytic process [48]. In contrast, Fructose-2,6-BP, a product of PFKFB3-catalyzed Fructose-6-P generation, was considered the most potent allosteric activator of PFK1, capable of stabilizing the PFK1 tetramer structure and counteracting the inhibition of PFK1 by high concentrations of ATP [49].

Cancer cells express different levels of PFK-2/FBPase-2 isozymes and regulate their relative kinase and bisphosphatase activities in a spatial or temporal manner according to metabolic needs [50]. PFKFB3 is widely overexpressed in OC, contributes to increased PFK1 activity, promotes aerobic glycolysis in OC cells, and is associated with poor patient prognosis [51]. Meanwhile, there is growing evidence that high levels of PFKFB3 are involved in chemoresistance in OC cells. Jiang et al. [52] demonstrated that PFKFB3 may enhance aerobic glycolysis and promote OC cell metastasis by regulating the apoptosis protein inhibitors and NF-κB signaling pathway. PFK-158 and 3-PO inhibit glycolysis by reducing glucose uptake, ATP production, and lactate metabolism through inhibition of PFKFB3 activity, which ameliorate resistance to carboplatin, paclitaxel and cisplatin in OC [52, 53]. In addition, knockdown of PFKP/PFKFB3 in OC-resistant cells also reduces levels of lactate and sensitizes the cells to cisplatin treatment, accompanied by increased poly ADP-ribose polymerase (PARP) cleavage [54]. It follows that PFKFB3 plays an important role in the regulation of glycolysis as well as growth and metastasis of OC. Therefore, targeting PFKFB3 to inhibit PFK1 activity could be a potential therapeutic option for OC. In addition, PFKFB2 also plays a role in OC development and chemoresistance. It was found that high CXCL14-expressing CAFs mediated the binding of LINC00092 to PFKFB2 in OC cells, thereby enhancing glycolysis of OC cells and the glycolytic phenotype of OC cells in turn contributed to the maintenance of local supportive functions of CAFs, enhancing the malignant behavior of the tumor [55]. Yang et al. [56] found that knockdown of PFKFB2, while increasing the rate of glycolysis, reduced the flow of intermediates through the pentose phosphate pathway in cancer cell lines with the wtTP53 gene, thereby decreasing NADPH levels, and that the accumulation of ROS after knockdown of PFKFB2 stimulated the phosphorylation of JNK, which induced G1 cell cycle arrest and apoptosis and reduced paclitaxel resistance. Similarly, PFKFB4 depletion enhances caspase 3/7 activity, increases ROS levels in OC cells during prolonged mitotic arrest, and promotes mitotic cell death after paclitaxel treatment [57]. Therefore, inhibitors of PFKFB2 or PFKFB4 in combination with paclitaxel may be an additional beneficial alternative for the treatment of OC.

### Pyruvate kinases, type M2(PKM2)

Pyruvate kinase is the last rate-limiting enzyme in the regulation of glycolytic metabolism and serves to catalyze the conversion of PEP and ADP to pyruvate and ATP. In mammals, the PK family consists of four isoforms: liver-type PK (PKL), red blood cell PK (PKR) and PK muscle isozyme M1 and M2 (PKM1 and PKM2, respectively) [58]. Among them, PKM2 showed a high level of expression in OC cells and a negative correlation with prognosis [59].

PKM2 exists as an inactive monomer, a less active dimer and an active tetramer. Among them, PKM2 tetramer increases glycolytic flux and promotes high ATP production, whereas dimeric PKM2 not only directly promotes aerobic glycolysis by redirecting glucose-generated carbon to biogenic enzymes, but also translocases to the nucleus and acts as a protein kinase to phosphorylate histone H3 and activate the transcription of various genes such as c-MYC and STAT3, which indirectly supports aerobic glycolysis and promoting the progression of tumor cell proliferation [60–63].

With regard to the regulation of OC pathogenesis by PKM2, Sun et al. [64] found that PSMD14 mediated the K63-linked deubiquitylation on PKM2, resulting in an increase in its dimerization ratio and nuclear translocation, which promoted aerobic glycolysis in OC cells, thereby stimulating the proliferation and invasion of OC cells. PKM2 could be targeted and upregulated by AKT2, which mediated elevated STAT3 expression and activation of nuclear translocation of NF-κB p65, increasing migration and invasion of OC cells in vitro [65]. Similarly, PKM2 expression could be upregulated by follicle stimulating hormone (FSH), which promoted aerobic glycolysis, and stimulated OC cell proliferation and cisplatin resistance [66]. In addition, PKM2 overexpression also affected the expression levels of several tumor-associated genes, such as up-regulation of CCND1 expression and reduction of CDKN1A expression, which increased the number of S-phase cells and induced the proliferation and survival of OC cells [67]. Whereas compound 3 K specifically inhibited pyruvate kinase M2, induced AMPK activation, accompanied by mTOR inhibition, which induced autophagic death of OC cells [68]. Based on these facts, blocking the glycolytic pathway and targeting cancer cell metabolism using specific PKM2 inhibitors may be a promising strategy for the treatment of OC.

## Regulatory mechanisms of aerobic glycolysis in OC

### AMP-activated protein kinase (AMPK)

The AMPK is a highly conserved Ser/Thr kinase, which is composed of catalytic α subunits and regulatory β and γ subunits, and serves as a well-known energy status sensor and regulator of energy homeostasis, encompassing glucose, protein and fatty acid metabolism, as well as autophagy [69, 70]. During energetic stress, AMPK inhibits ATP consuming processes, such as fatty acid and protein synthesis, and cell proliferation, while promoting ATP preservation processes, including autophagy, glycolysis, TCA cycle and fatty acid oxidation [71]. AMPK is commonly activated during energetic stress by upstream kinases, including liver kinase B1 (LKB1) and Ca^2+^/calmodulin-dependent protein kinase kinase (CaMMKβ), which mediate the phosphorylation of threonine 172 (Thr^172^) in the catalytic α subunit of AMPK [72].

A growing number of studies have proved that AMPK plays an essential role in the proliferation, metastasis and invasion, and stress response in OC. Firstly, it has been found that transient receptor potential 7 (TRPM7) silencing activates the AMPK signaling pathway and promotes the ubiquitination degradation of HIF-1α, thereby inhibiting OC cell glycolysis and proliferation [73]; whereas inhibited AMPK activity is positively correlated with the increased aggressiveness of OC cells, thereby leading to a deteriorated prognosis for patients [74]. Secondly, it has also been suggested that AMPK may act as a tumor promoter related to the LKB1-AMPK pathway. For example, this pathway stabilizes the formation of epithelial OC cell spheroids, which promoted the development of metastasis and peritoneal lesions [75]. And under energy stress, lipolysis-stimulated lipoprotein receptor (LSR) also increases the phosphorylation levels of AMPKα and acetyl-CoA carboxylase (ACC) through this pathway, thereby promoting cancer cell survival and tumor progression [76]. Thirdly, AMPK also connects metabolic and signaling pathways, and its activation under high-energy stress maintains the link between glucose metabolism and cancer progression through GLUT3-mediated glucose energy homeostasis and regulation of the Hippo pathway [77].

In conclusion, AMPK represents a promising target for OC treatment. It has been discovered that natural AMPK activators, such as bitter melon extract, resveratrol, cordycepin, honokiol, or the novel AMPK-activating compound NT1014 may present new treatment options for OC by either inhibiting cell proliferation or promoting autophagy-induced cell death [78–82].

### PI3K/AKT pathway

The phosphoinositide 3-kinases (PI3Ks) are lipid kinases that convert extracellular stimuli into intracellular signals through the production of phosphatidylinositol-3,4,5-trisphosphate (PIP3). Based on their different structures and specific substrates, PI3Ks are divided into three classes: class I (including class Ia and Ib), class II and class III [83]. Akt is a key serine/threonine kinase involved in the PI3K signaling pathway [84]. The PI3K/AKT signaling pathway broadly regulates normal cellular processes, but it is altered in cancer cells, promoting their proliferation, growth, motility, metabolism, angiogenesis, and inflammatory responses, and affecting the tumor microenvironment [85]. Thus, in OC, cell viability and EMT were similarly impaired after inhibition of the PI3K/AKT pathway by miR-22 [86]. PI3K/Akt pathway activation also contributes to cisplatin resistance in OC cells [87]. Using LY294002, a PI3K/Akt signaling pathway inhibitor could reverse cisplatin resistance in OC cells [88]. In addition, PTEN, as an oncogene, could induce dephosphorylation of PIP3, prevent Akt activation, and play a negative regulatory role in the PI3K/Akt signaling pathway, thus inhibiting tumor growth [89]. Compared with normal ovarian tissues, PTEN expression was absent in ovarian malignant tumor tissues, and PTEN expression in tissues was positively correlated with survival time and prognosis of patients [90].

The PI3K/AKT signaling pathway regulates glycolysis of OC through various mechanisms. Firstly, the PI3K/Akt signaling pathway induces the expression of GLUT1 and GLUT3, and mediates the translocation of GLUT4 from the cytoplasm to the cell membrane to enhance glucose uptake and transport [91]. Secondly, PI3K/Akt regulates the activity and expression of some glycolytic enzymes: (1) The oncogene ACTL6A accelerates OC glycolysis by up-regulating the PI3K/Akt pathway via FSH, which in turn regulates PGK1 to promote glucose utilization, lactate production, and pyruvate levels [24]. (2) The expression of HK2 can be up-regulated by the PI3K/AKT pathway, and the oncogene ARHGAP10 can inhibit OC cell glycolysis through this pathway [39]. (3) PKM2 can be regulated by epidermal growth factor (EGF) through the PI3K/AKT2 pathway, and the PI3K inhibitor LY294002 can block this effect [65]. Thirdly, PI3K/AKT indirectly affects glycolytic flux by interacting to regulate AMPK and HIF expression. For example, the PI3K/Akt pathway and the AMPK pathway can be synergistically affected by the enhanced p21 expression after creatine kinase B knockdown, resulting in glycolysis inhibition and OC apoptosis [92]. Additionally, knockdown of HIF-1α inhibited the PI3K/Akt/mTOR pathway and promoted autophagy in OC cells [93]. Finally, PI3K/AKT activates downstream regulators such as mTOR and VEGF to promote glycolysis and angiogenesis in OC. Wang et al. [94] found that FBN1 mediated the phosphorylation of vascular endothelial growth factor receptor 2 (VEGFR2) and activated the AKT pathway, which in turn induced the phosphorylation and nuclear translocation of STAT2, promoting glycolysis and angiogenesis. In addition, integrin alpha x (ITGAX) upregulates VEGFR2/VEGF-A expression through the PI3K/Akt pathway, which further promotes angiogenesis and OC progression [95].

### Noncoding RNAs

Non-coding RNAs (ncRNAs) account for more than 90% of the human genome, which cannot be transcribed into proteins but are involved as functional RNAs in a variety of biological processes including development, proliferation, post-transcriptional modification, apoptosis, and cellular metabolism. ncRNAs can be mainly classified into three categories: long noncoding RNA (lncRNA), microRNA (miRNA), and circular RNA (circRNA), which can influence the “Warburg effect” by regulating the expression of glycolytic enzymes or glycolysis-related pathways [96, 97].

The three rate-limiting enzymes in aerobic glycolysis can all be regulated by various noncoding RNAs (Table 1). For example, the miR-654-3p was found to be inhibited by circular RNA pyridoxal kinase (circPDXK), which was highly expressed in tumor tissues and cells of OC patients, leading to upregulation of HK2 expression [98]. The miR-200 positively regulates HK2, which was associated with the fact that WTAP (WT1-associated protein) expression could be upregulated by HIF-1α and WTAP was able to interact with DGCR8 [99]. In addition, the DNMT3A/miR-603/HK2 axis was also involved in the “Warburg effect”, and the miR-603 expression was inhibited by DNMT3A, which promoted cell proliferation, migration, and invasion in OC [100]. Another key enzyme for glycolysis, PKM2, was found to be directly targeted by miR-324-5p, which negatively regulated glycolysis and lactate production in OC cells [101]. LINC00504 was up-regulated in OC tissues and was reported to inhibit miR-1244 to enhance the expression of glycolytic enzymes, including HK2, PKM2, and PDK1 [102]. By binding to PFKFB2, LINC00092 altered glycolysis and maintained local supportive functions of CAFs to promote metastasis of OC [55]. While the miR-206 directly targeted to regulate PFKFB3 expression and influenced glycolysis of OC [103]. In addition, lncRNA ceruloplasmin (NRCP) could function as an intermediate binding chaperone between STAT1 and RNA polymerase II, leading to increased expression of glucose-6-phosphate isomerase (G6PI) [104].

**Table 1:** Noncoding RNAs and their targets in aerobic glycolysis in OC

	Target	ncRNA	Involvement of other factors	Reference
Glucose uptake	GLUT1	miR-1204	—	^101^
Glycolytic enzymes	HK2	miR-654-3p	circPDXK	^93^
		miR-200	HIF-1α/WTAP/ DGCR8	^94^
		miR-603	DNMT3A	^95^
		LINC00504	miR-1244	^97^
	PKM2	miR-324-5p	H19	^96^
		LINC00504	miR-1244	^97^
	PDK1	LINC00504	miR-1244	^97^
	PFKFB2	LINC00092	CXCL14/CAFs	^98^
	PFKFB3	miR-206	FAK	^99^
	G6PI	NRCP	STAT1	^100^
	LDHA	LncRNA HOXB-AS3	miR-378a-3p	^102^
AMPK pathway	LKB1/AMPK/TORC2	miR-3652	SIK/TORC	^103^
Wnt pathway	SFRP1/Wnt	miR-1180	—	^104^
MYC pathway	c-MYC	LINC00629	HOXB4	^105^
		LncRNA CTSLP8	PKM2	^106^
Hippo pathway	Hippo/YAP1	LINC00857	miR-486-5p	^107^
PI3K/Akt pathway	PI3K/AKT	circRHOBTB3	—	^108^
	AKT2/AKT3	miR-29b	HK2/PKM2	^109^
HIF-1 pathway	HIF-1α	LINC00662	miR-375	^110^

Other key proteins such as GLUT1 and LDHA are also regulated by ncRNAs in the OC, leading to changes in the levels of glucose uptake and lactate production. For example, the expression level of miR-1204 was positively correlated with that of GLUT1 in OC patients, and the overexpression of miR-1204 significantly promoted GLUT1 expression, enhanced glucose uptake and cell proliferation [105]. In addition, lncRNA HOXB-AS3 was abundantly expressed in EOC tissues, which was tightly associated with unfavorable overall survival status of the patients, whereas knockdown of HOXB-AS3 reduced LDHA expression via sponge miR-378a-3p, affecting the rate of acidification in the TME [106].

Non-coding RNAs can also regulate glycolysis levels in OC by targeting various signaling pathways involved in glycolysis. For example, downregulation of miR-3652 expression enabled overexpression of the CRTC2 gene, which functioned in the SIK/TORC pathway and was involved in the regulation of glucose production through the LKB1/AMPK/TORC2 pathway [107]. The miR-1180 activated Wnt signaling by targeting SFRP1 in OC cells to accelerate their proliferation and glycolysis [108]. The c-MYC is the target of LINC00629 and lncRNA CTSLP8 [109, 110], and the Hippo signaling pathway is the target of lncRNA LINC00857 [111], and PI3K/AKT is the target of circRHOBTB3 and miR-29b [112, 113]. Through directly binding to miR-375, LINC00662 was able to act on HIF-1α to promote glycolysis of OC and reduce apoptosis [114].

### The role of glycolysis in peritoneal metastasis in OC

Altered glycolysis in cancer cells and related cells in the metastatic environment, such as mesothelial cells and cancer-associated fibroblasts(CAFs), can promote the process of peritoneal metastasis in OC.

Overexpressed in OC, especially in ascites and metastatic foci, HK2 can control lactate production and promote the process of OC metastasis via FAK/ERK/1/2 signaling pathway-mediated expression of MMP9/NANOG/SOX9 [115]. Knockdown of the HK2 gene in OC cells and ascites-derived tumor cells with the use of the glycolysis inhibitor 2-DG impeded lactate production, cell migration and invasion, and cell stemness properties, while decreasing FAK/ERK1/2 activation and metastasis and stemness-related genes [116]. Increased levels of fatty acid synthase correlate with high expression of P-cadherin, supporting enhanced ab initio lipogenesis in the metastatic microenvironment. P-calmodulin may enhance the activity of mesothelial cell glycolytic enzymes HK2, PGK1 through signaling cascades involving HIF or AMPK [117], thereby promoting glycolysis under metabolic stress or hypoxic conditions. Enriched lactate produced by the glycolytic process provides substrates for lipid synthesis and promotes ab initio synthesis of lipids in highly metastatic OC. Inhibition of lactate uptake by targeted inhibition of MCT1 could eliminate metabolic crosstalk and mesothelial metastasis [118]. Furthermore, PFKFB2 expression was significantly higher in metastatic OC tissues than in normal ovarian tissues and OC tissues without metastasis. It has been mentioned previously that binding of PFKFB2 to LINC00092 maintains the glycolytic phenotype of OC cells and enhances tumor aggressiveness through interactions with CAFs that highly express CXCL14 [55]. Schab et al. [119] found that DDR2 is highly expressed in OC stromal cells, which inhibits fructose 1,6-bisphosphatase and increases the activity of the key enzyme HK by targeting the AKT/SNAI1 pathway and that DDR2 increases the secretion of the collagen cross-linking agent LOXL2, which promotes the secretion of extracellular matrix proteins, and enhances the ability of cancer cells to spread in the peritoneal cavity.

### Glycolysis mediates immunosuppression

**Fig. 3:**
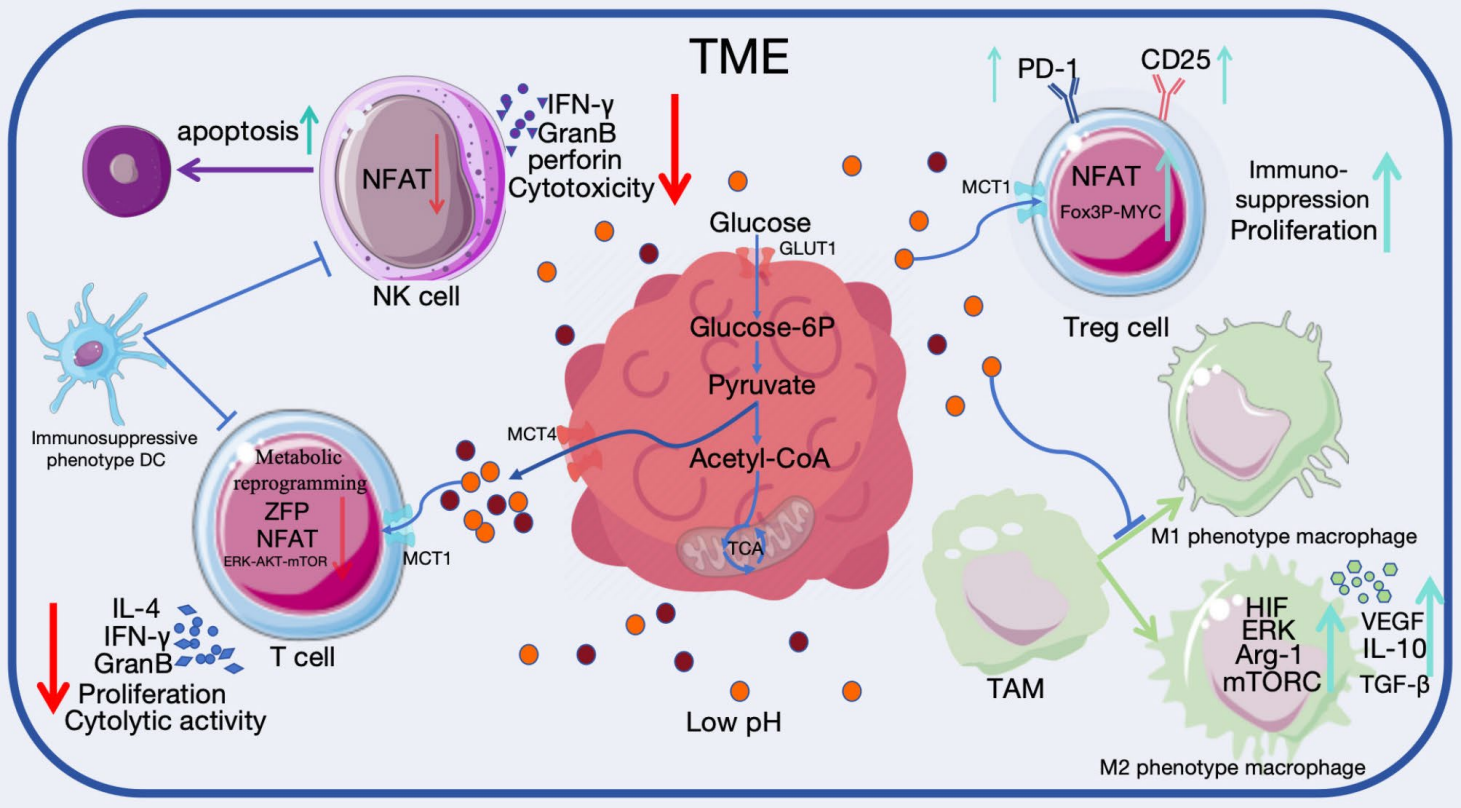
Impact of lactate accumulation on immune cells within the TME. Tumor cells produce excess lactate through aerobic glycolysis, causing acidosis and immunosuppression. Tumor-derived lactate regulates immune cell metabolism, inhibits the activation and proliferation of immune cells such as T cells, macrophages, and NK cells, and acts as a signaling molecule to regulate the immune response of tumor cells. In contrast, Treg cells and M2 phenotype macrophages are better able to maintain their immunosuppressive function in the acidic TME caused by lactate

Lactate produced by glycolysis in cancer cells is one of the important metabolites in the tumor microenvironment, which is closely related to the immune escape of tumor cells. It can regulate the metabolism of immune cells, inhibit the activation and proliferation of immune cells such as T cells, macrophages, natural killer cells (NK cells) and dendritic cells (DCs), and regulate the immune response of tumor cells as a signal molecule, which plays an essential role in immune surveillance and escape [120, 121]. This new research direction has a variety of potential clinical applications. Therefore, here we will focus on the research progress of the regulation of lactate on T cells, NK cells, Treg cells, tumor-associated macrophages (TAMs), which promotes immune escape of cancer cells (Fig. 3).

### T cells

T cell-mediated immune responses play an essential role in inhibiting tumor cell growth. T cells involved in tumor immunity include MHC class I antigen-restricted CD8^+^ cytotoxic T lymphocyte (CTL), MHC class II antigen-restricted CD4^+^ helper T cell (TH), tumor-promoting Tregs and etc. T cells are capable of sensing extracellular lactate levels, which elicits intracellular adjustments in signaling, cellular function, and homeostasis of the internal environment. Acidosis caused by excess extracellular lactate inhibits T cell-mediated immune responses, and tumor-specific CD8^+^ T cells are usually induced to an unresponsive state with reduced cytolytic activity and cytokine production at extracellular environmental pH values of 6.0 to 6.5 [122, 123]. In addition, lactate affects the metabolic aspects of T cells, which can inhibit the proliferation and cytokine production of human CTL with an efficiency of up to 95% and leading to a 50% reduction in cytotoxicity, which is attributed to the fact that high extracellular concentrations of lactate prevent the excretion of lactate from the cytoplasm of CTL, disrupting the metabolism of CTL [124]. In a high lactate environment, the lactate concentration difference formed inside and outside the cell prompts extracellular lactate to enter the cell by diffusion through MCT1 or other transporters on T cells as well as transporter-independent diffusion, leading to an intracellular low pH environment that prevents the upregulation of nuclear factor of activated T cells (NFAT) in T and NK cells, which leads to the reduction of interferon-γ (IFN-γ) and affects the activation of T cells and the participation in the process of tumor immunosurveillance. At the same time, intracellular acidification reduces the production of granzyme B by T cells and NK cells, which weakens the cytotoxicity and fails to target the apoptosis of tumor cells [125]. In addition, tumor cell-derived lactate impairs the function of natural killer T cells by inhibiting the mTOR signaling pathway and nuclear translocation of zinc finger proteins, resulting in reduced secretion of IFN-γ and IL-4 and significantly weakened anti-tumor effect [126]. The increased extracellular acidification rate (ECAR) of glycolytic phenotype OC stem cells attenuates T cell responses to TCR stimulation and reprograms T cell metabolism to inhibit T cell activation, proliferation, and action, as well as suppressing T cell responses by inhibiting ERK-AKT-mTOR signaling and activating the integrated stress response pathway [127]. In addition to its effects on common types of T cells, lactate promotes loss of the FAK family-interacting protein FIP200 in OC, driving apoptosis of naïve T cells and decreases cholesterol synthesis and IFN-γ release from invariant natural killer T cells (iNKT cells), which ultimately affects tumor immunity [128, 129].

Understanding the mechanisms by which the tumor microenvironment regulates T cells is of critical importance for the use of immunotherapy against cancer. However, there are still many unknowns in this complex microenvironment that are waiting to be explored.

### NK cells

NK cells, as an effective component of tumor immune surveillance and control, have a favorable prognostic impact on OC [130]. NK cells, the main effectors of the innate immune system, produce antibody-dependent cytotoxicity as well as granzymes and perforins on target cells lacking MHC class I molecules when activated, as well as apoptotic signaling of target cells through the Fas/FasL and TRAIL/TRAILR pathways and secretion of a variety of cytokines including TNF-α and IFN-γ, which kill OC cells directly or indirectly participate in the immune response against OC development [131, 132]. NK cells can recognize stress-associated antigens through the NKG2D receptor, and this recognition is MHC-independent. MICA is a functional ligand that stimulates the NKG2D receptor, and in addition to MICA, the interaction of MICB and ULBP, which are part of the human NKG2D ligand, with the NKG2D receptor is important for cancer cell recognition and NK cell-mediated cytotoxicity [133]. Studies have shown that MICA/B and ULBP2 are expressed in most OC cells, but not in normal ovarian epithelium. Human OC over-expresses a variety of matrix metalloproteinases, such as MMP2, MMP9 and MMP14. MICA/B and ULBP2 on the membrane of OC cells can be cleaved by matrix metalloproteinases to produce soluble molecules, which systematically downregulate the expression of NKG2D receptor or directly block the function of NKG2D, thus impairing the immune surveillance of NK cells, resulting in the immune escape of OC cells [134–136]. The anti-stress natural product Ashwagandha is able to block tumor-induced NK cell inhibition by down-regulating the expression of GRP78 and the protein hydrolyzing agent ADAM10 on the surface of OC cells, thereby reducing MICA/B shedding [137]. In addition, OC-associated miR-20a was found to bind directly to the 3’-untranslated region of MICA/B mRNA, leading to the degradation of MICA/B and a decrease in the level of MICA/B proteins on the OC cell membrane, which prevented NK cells from exerting cytotoxicity against OC [138].

Lactate, as an important participant in suppressing tumor immunity, also has a significant impact on NK cells. The presence of lactate in the TME leads to polarization of the immunosuppressive phenotype of dendritic cells, thereby impairing the cytotoxicity of T and NK cells [139]. The key enzyme of lactate production, LDH, can spontaneously release its activity, a feature that is related to the dysfunction of NK cells. The acidic environment caused by lactate in tumor tissues can significantly inhibit the activity of NK cells and significantly reduce the release of cytotoxic molecules, such as perforin and granzymes, resulting in a reduction of their killing effect on tumor cells, and tumor-derived lactate can induce apoptosis of NK cells and reduce their infiltration in vivo [125, 139]. Large amounts of lactate can lower the pH in the tumor microenvironment, which in turn prevents NK cells from expelling cytoplasmic lactate down the lactate concentration gradient, leading to impaired metabolism, mitochondrial stress and apoptosis in NK cells [140]. Neutralization of the acidic environment in tumor tissues or inhibition of MCT4 in tumor cells to inhibit the release of lactate into the extracellular environment can restore the activity of NK cells in the TME to a certain extent [141]. In conclusion, it is possible that the inhibition of NK cell activity by lactate can be reversed by relevant treatment.

### Treg cells

Treg cells can inhibit the abnormal over-immunization of immune cells against antigens, thus maintaining the immune homeostasis of the body, and they generally inhibit anti-tumor immunity and play a pro-tumorigenic role. For example, in vitro-induced CD8^+^ Treg cells inhibit the proliferation of naïve CD4^+^ T cells through TGF-β1 and IFN-γ mediation [142]. Alternatively, CD4^+^ T cells can be promoted for conversion to Treg cells by immunosuppressive regulatory B cells that produce IL-10 and express high levels of CD80 and CD86, thereby inhibiting CD4^+^ and CD8^+^ T cell proliferation [143].

Unlike other immune cells that show a negative response to lactate, Treg cells are able to increase their infiltration within tumors in an environment overwhelmed with lactate and upregulate the Treg cells activation marker CD25, and lactate released by tumor cells in the TME impairs cytotoxic T cell function and causes apoptosis, but Treg cells are highly resistant to this, and lactate can be used as an energy source for their uptake and utilization, as well as supporting more active metabolism of Treg cells [144, 145]. In the TME of low glucose and high lactate, forkhead frame protein 3 (FOXP3) in Treg cells, as a metabolic regulatory hub, has the ability to down-regulate the level of cellular glycolysis and induce OXPHOS by modulating MYC gene expression, thus increasing the ratio of NAD^+^ to NADH in the cells. High concentrations of lactate damage effector T cells through LDH-mediated NADH depletion, but Treg cells are highly resistant to it, which in turn promotes tumor development [146]. Compared to patients with benign ovarian tumors and healthy controls, OC patients had an increased CD8^+^ Treg cell subset with increased expression of CD25, CTLA-4, and Foxp3 and decreased expression of CD28, whereas CD8^+^ Treg cells induced in vitro by co-culture with OC cells similarly showed increased expression of CTLA-4 and Foxp3 and decreased expression of CD28 [142]. In addition, the large amount of lactate taken up by Treg cells can also be used for their own gene expression regulation and epigenetic modification. Lactate-modified MOESIN is able to promote the activation of the TGF-β-mediated SMAD3 signaling pathway and the transcription of the FOXP3 gene, which can enhance the function of Treg cells and drive the tumorigenesis [147]. Around highly glycolyzed tumor cells, Treg cells actively take up lactate via MCT1, which promotes NFAT1 into the nucleus, thus enhancing PD-1 expression, while PD-1 expression of effector T cells is inhibited, resulting in Treg cells expressing more PD-1 than effector T cells, leading to a decreased anti-PD-1 therapeutic effect [148]. In addition, mTOR and its downstream signaling pathway were significantly activated in CD4^+^ Treg of OC patients. mTOR signaling pathway could affect the glucose metabolism process of CD4^+^ Treg by regulating the levels of CD4^+^ Treg’s glucose uptake and glycolysis, and modulating the expression levels of glucose metabolism-related genes and proteins. Inhibition of mTOR signaling in CD4^+^ Tregs in OC cells growth environment resulted in a significant decrease in the expression levels of glucose metabolism-related genes and proteins, as well as a significant decrease in the levels of glucose uptake and glycolysis, and the simultaneous inhibition of mTOR signaling and activation of TLR8 signaling had a synergistic inhibitory effect on glucose metabolism and the immune-suppressing function of CD4^+^ Tregs. Activation of TLR8 signaling inhibited glucose metabolism in CD4^+^ Tregs by down-regulating mTOR signaling, thereby reversing the immunosuppressive function of these cells in the OC cells growth environment [149–151]. Gao et al. [152] found that miR-124 reduces lactate uptake by directly targeting MCT1, which ultimately impairs the immunosuppressive function of Treg cells, thereby slowing down the growth of OC cells and increasing their response to PD-1 blocking therapies. Wang et al. [153] demonstrated that oridonin could inhibit the metastasis of OC cells by inhibiting the mTOR signaling pathway and upregulating the level of FOXP3. Therefore, it is evident that targeting inhibition of MCT1, mTOR signaling and activation of TLR8 signaling in tumor tissues enriched with Treg cells may contribute to the improvement of immunotherapy efficacy of OC.

### TAMs

Macrophages are one of the most infiltrated immune cell populations within the TME in OC and are mainly differentiated from monocytes, including M1 phenotype and M2 phenotype. Macrophages themselves have strong plasticity, and M1 phenotype macrophages produce inflammatory and pro-Th1 cytokines and low concentrations of IL-10, which have tumor-killing effects, while M2 phenotype polarized macrophages secrete a variety of anti-inflammatory factors and growth factors, which have immune-suppressive functions and play a key role in tumor development [154, 155]. Compared with specimens from benign ovarian lesions, patients with OC had increased densities of TAMs, more pronounced infiltration, and decreased M1/M2 ratios in their cancer tissues, thus exerting a role in promoting tumor growth, invasion, and metastasis, and suggesting a poor prognosis [156, 157]. Unlike other immune cells, the interaction between lactate and TAMs is reciprocal. On the one hand, there is evidence that the large amount of lactate in the TME significantly affects the polarization phenotype of macrophages, and that tumor cell-derived lactate induces polarization toward the M2 phenotype by stabilizing HIF-1α and promoting angiogenesis-induced production of VEGF, which phenotype demonstrates greater survival and adaptive capacity than M1 phenotype in the acidic environment of tumor tissues [158, 159]. Besides, OC-derived lactic acidosis promotes differentiation of monocytes into M2 phenotype macrophages with high levels of CD14 and CD163 markers and IL-1β, and certain OC cell lines generate IL-6 and M-CSF in addition to lactate to induce M2 phenotype macrophage polarization [160]. It was found that mTORC-related signaling pathway activation is essential in lactate-mediated M2 phenotype polarization of macrophages. Lactate activates the mTORC1 signaling pathway in TAMs, which in turn downregulates the expression of the lysosomal vesicle hydrolase subunit ATP6V0d2 through transcriptional regulation, and macrophage intracellular HIF-2α protein stability is increased as a result of lysosomal functional limitation, which ultimately maintains its own tumor-promoting M2 phenotype and supports tumor progression [161]. Chen et al. [162] found that the phosphorylation of mTOR, signal transduction and STAT3 could be inhibited by cardamonin, which exerted the effect of inhibiting M2 phenotype macrophage polarization and down-regulating the secretion of oncogenic factors by TAMs, and thus impeded the tumor-promoting function of TAMs on OC cells. The TAM surface receptor Gpr132 functions in response to lactate stimulation and induces its own M2 phenotype polarization, whereas the tumor-associated macrophage surface olfactory receptor Olfr78 has a similar effect. Olfr78 synergistically mediates the polarization of M2 phenotype macrophages with Gpr132, and Olfr78 functionally deficient mice exhibit the characteristics of tumor growth suppression, metastasis inhibition, and increased anti-tumor immune cell populations [163]. On the other hand, TAMs also affect tumor cell glycolytic metabolism as well as promote tumor progression. Colegio et al. [158] found that tumor-derived lactate was able to induce M2 phenotype polarization of macrophages via HIF-1α as well as arginase 1(Arg 1), while polarized macrophages could increase the utilization of lactate. In addition, lactate produced by tumors stimulates the production of IL-23 in TAM, leading to tumor growth by inducing the production of IL-17 and IL-22 [154]. Overall, the interactions between tumor cells and macrophages in the TME play a crucial role in the metabolic changes of macrophages, and lactate in the TME promotes their metabolic reprogramming, prompting macrophages to undergo an M2 phenotypic switch, in which the M1 phenotype relies mainly on aerobic glycolysis and fatty acid synthesis, whereas the M2 phenotype macrophage relies more on OXPHOS and fatty acid oxidation, and this phenotypic transition profoundly affects the TAMs immune response to tumors [154]. Additionally, it has been observed that OC cells are able to secrete lactate to promote the synthesis and secretion of IL-1β by TAMs, and the secreted IL-1β activates the nuclear factor kappa-B (NF-κB) signaling pathway in cancer cells to induce the up-regulation of the expression of PD-L1 and CCL2. IL-1β not only leads to the inhibitory effect of cancer cells on CD8^+^ T cells directly, but also recruits other components of immunosuppressive cells, including M2 phenotype macrophages, into the microenvironment, generating a vicious cycle leading to tumor immune escape. Targeting IL-1β can reverse the immunosuppressive phenotype of the TME, and its synergistic effect with PD-1-targeted immune checkpoint therapy can be achieved [164].

### Endothelial cells and dendritic cells

Endothelial cells (ECs) in the TME are an integral part of the vasculature and are critical in transporting substances, angiogenesis and tumor metastasis. ECs in tumor tissues show significant heterogeneity and plasticity, which express lower levels of adhesion molecules, leading to impaired barrier function and increased levels of inhibitory immune checkpoint molecules which contribute to immunosuppression [165]. Like OC cells, ECs in cancerous tissues are more dependent on glycolysis for energy production than normal ECs, exhibiting a hyper glycolytic phenotype, the mechanism of action of which may be related to increased expression of GLUT1 and the glycolytic metabolizing enzyme 6-phosphofructo-2-kinase/fructose-2,6-biphosphatase 3 (PFKFB3) [103]. Hypoxia and pro-inflammatory cytokines in the TME upregulate PFKFB3. It has been demonstrated that inhibition of PFKFB3 reduces glycolysis in ECs in the TME to prevent the proliferation of tumor cells. High doses of 3-PO, a small molecule inhibitor of PFKFB3, can inhibit proliferation of cancer cells and reduce the growth of primary tumors, whereas a low dose of 3-PO induced tumor vascular barrier tightening and maturation, reducing cancer cell intravasation and metastasis [166]. Besides, lactate released into the microenvironment by tumor cells demonstrates the function of activating vascular ECs in tumor tissues and promoting angiogenesis. The uptake of lactate by ECs via MCT1 into the intracellular compartment for conversion to pyruvate results in the accumulation of NADH, which activates NADH oxidase, thereby inducing an increase in ROS and inhibition of prolyl hydroxylase domain 2 (PHD2). The increase in ROS and the inhibition of PHD2 lead to an increase in the degradation of human nuclear factor kappa-B inhibitor alpha (IκBα), which promotes the activity of NF-κB and activates the pro-angiogenic signaling IL-8 pathway to achieve the goal of promoting blood vessel growth [167].

Monocyte-derived dendritic cells exhibit an essential role in differentiation and activation of T cells, and normal functioning DCs must be available in tissues to serve as antigen-presenting cells, which recognize and process tumor antigens, form antigen-MHC complexes that are expressed on the surface of the cells for T-lymphocytes to recognize, promoting the maturation of naive T-lymphocytes. However, lactate-mediated signaling blocks the normal differentiation of DCs and affects their activation and antigen presentation, resulting in a tendency towards an immunosuppressive phenotype, in which DCs have lower levels of MHC II and co-stimulatory molecules, produce lower levels of IL-12 and higher levels of IL-10, and reduce the production of immune-activating cytokines such as IFN-γ, TNF-α, IL-1β and IL-6, which prevent the presentation of cancer cell-specific antigens to immune cells, negatively regulate the immune response, and result in immunosuppressive effects [168, 169]. Additionally, lactate induces intracellular Ca^2+^ mobilization by binding to GPR81 on the surface of plasmacytoid dendritic cells (pDCs) or transported to the cytoplasm of pDCs via MCT1, which participates in calcineurin phosphatase (CALN) signaling and affects cellular metabolism required for pDCs activation, and attenuates release of IFN-α by pDCs, affecting anti-tumor immune responses, while elevated intracellular lactate increases tryptophan metabolism in pDCs, leading to overproduction of L-kynurenine, which contributes to the expansion of FoxP3^+^CD4^+^ regulatory T cells, the main immunosuppressive immune cell subset in the TME [170]. Moreover, pDCs can secrete high levels of IFN-α through TLR, but the functional activity of pDCs in the acidic TME was altered, and instead, they highly expressed immunosuppressive factors such as IL-10, TGF-β, and indoleamine 2,3-dioxygenase (IDO), which induced the expansion of Tregs. Meanwhile, IL-10 enhances the secretion of serine protease granzyme B (GrB) from pDCs and inhibits the expansion of CD4^+^ T cells [171, 172]. The various effects of lactic acid accumulation on DCs play a negative role in the first stage of tumor cellular immunity, i.e., the antigen presentation process, thus impeding tumor immunity. Therefore, immunotherapy aimed at enhancing the antigen presentation function of DCs, inducing or enhancing the effective anti-tumor immune response against tumor antigens would be a very effective anticancer means.

### Targeting glycolysis combined with immunotherapy in OC

TME is increasingly recognized as a key factor in tumor progression, and although researches on the effects of TME on immunotherapy and the mechanisms of its regulation are still in progress, its presence of negative regulation of immunotherapy cannot be ignored. In tumor tissues, the metabolites present in TME cause inhibition of immune cell function, especially contributing to the immune deficiency of tumor-infiltrating T lymphocytes. Therefore, interventions targeting glucose metabolism of cells in the TME to influence the composition of components in the TME become attractive adjunctive therapies to synergistically improve the anti-tumor immune response induced by the immune checkpoint inhibitor (ICI). Targeted understanding the effects of different metabolites on other immune cell components could also create more selective solution strategies for cancer immunotherapy (Table 2).

**Table 2:** Compounds and their targets and experimental phases in OC therapy

Compounds	Target	Involvement of other factors	Experimental stage	References
PSVII	GLUT1	RORC/ACK1	In vitro and in vivo	18
	HK2			
NT1014	PDH	AMPK/mTOR	In vitro and in vivo	82
	LDHA			
LY294002	PKM2	EGF/PI3K/AKT2	In vitro and in vivo	65
BAY-876	GLUT1	AMPK	In vitro and in vivo	174
2-DG	HK2	FAK/ERK1/2	In vitro and in vivo	116
DCA	PDK1	PD-1/PD-L1	In vitro and in vivo	175
AZD3965	MCT1	PD-1/PD-L1	In vivo	179
MLN0128	TORC1/2	/	Phase Ib	182
PP242	TORC1/2	AKT	In vitro and in vivo	183
ATG-008	TORC1/2	PD-1/PD-L1	Phase 1/2	181
AZD2014	TORC1/2	/	In vitro and in vivo	184
NVP-BEZ235	PI3K/mTOR	AKT	In vitro	185

Reducing glycolytic metabolism in cancer cells by inhibiting key enzymes of glycolysis or focusing on the use of the competitive glucose analog 2-DG or the GLUT1 inhibitor BAY-876 is effective in inhibiting cancer cell proliferation, which may be able to support the formation of long-term memory CD8^+^ T cells [173, 174]. It was found that the glycolysis gating enzyme PDK1 in OC inhibits IFN-γ secretion by over-activating the PD-1/PD-L1 pathway through the c-Jun-NH2-kinase (JNK)-c-Jun pathway, resulting in the inhibition of CD8^+^ T cell function, and the knockdown of PDK1 in OC cells was able to block its induced expression of PD-L1 and promote the activation and infiltration of CD8^+^ T cells into tumor tissues to inhibit tumor growth, and the combination of PDK inhibitor dichloroacetic acid (DCA) and PD-L1 antibody increased IFN-γ secretion in monocyte-infiltrated tumor islets, attenuated tumor immune escape, and significantly enhanced the anti-tumor effect of immune checkpoint blockade [175]. Besides, Ho et al. [176] found that overexpression of the gluconeogenesis enzyme PCK1 enhanced the antitumoral response of T cells in glucose-deficient TME by inhibiting sarco/ER Ca^2+^-ATPase (SERCA) activity, and the results of this study support that glycolytic intermediates play an important role in the proliferation and function of effector T cells. Further, it has been shown that immune checkpoints including PD-1, PD-L1, and CTLA-4 act by partially inhibiting metabolic reprogramming and glycolysis of immune cells while increasing lipolysis and fatty acid β-oxidation (FAO) [177]. These studies reveal a link between cellular metabolism and checkpoint blockade, and thus approaches combined with alterations of glucose metabolism in cancer cell or immune cell may provide new opportunities for immunotherapy and improve the efficacy of checkpoint inhibitors in OC.

The aerobic glycolysis in cancer cells makes lactate prevalent in the TME, which is detrimental to antitumoral immunotherapy. GAO et al. [178] constructed a hollow MnO2 (HMnO2)-catalyzed nanosystem (PMLR) loaded with lactate oxidase (LOX) and glycolysis inhibitor (3-PO) and encapsulated in red blood cell membranes (mRBC). Owing to the long-circulating properties of mRBC, PMLR gradually accumulates at the tumor site, and the extracellular PMLR catalyzes the oxidative reaction of lactate via LOX to consume the lactate in TME, while intracellular PMLR releases glycolysis inhibitors to block the lactate source. Moreover, PMLR decreased the proportion of M2 phenotype macrophages in combination with PD-L1 therapy, and immune-activating cytokines, IFN-γ and IL-6, were detected, and the survival of experimental mice was prolonged compared with PD-L1 therapy alone, suggesting that lactate depletion can improve the efficacy of PD-L1 therapy. In addition, inhibition of MCT function to disrupt lactate transport between cells in tumor tissues also plays a role in suppressing tumor development. For lactate produced by high flux of glycolysis in cancer cells, an acidic environment-activatable nanomedicine, AZD3965, was able to reverse lactate-induced tumor immunosuppression by targeting on inhibiting MCT1 expression. Moreover, when oral AZD3965 is combined with anti-PD-1 therapy, increased T cell infiltration and decreased depletion of PD-1^+^ Tim3^+^ T lymphocyte effectively inhibits tumor growth and improves survival [179]. Additionally, given the important role of the mTOR pathway in lactate metabolism, combination therapy with mTOR inhibitors and lactate metabolism or transport inhibitors is considered to enhance anti-tumor immunity. Rapalogs (temsirolimus, everolimus), ATP-competitive mTOR inhibitors (MLN0128, PP242, AZD2014, AZD8055) and dual PI3K/mTOR inhibitors (NVP- BEZ235, LY3023414, etc.) have been applied to treat a variety of cancers, and the mTORC1/2 inhibitor onatasertib (ATG-008) demonstrates synergistic anti-tumor activity in combination with PD-1 antibody [180, 181].

To date, fewer studies have been conducted on the combined use of glycolytic or lactate inhibitors and immunotherapy aimed at OC, and further studies are expected to clarify the role of cancer-specific glycolytic inhibitors in combination with immunotherapy in the creation of new effective therapeutic regimens in OC. 

## Conclusion

A defining characteristic of OC is the alteration in metabolic processes, specifically aerobic glycolysis. This phenomenon provides valuable insights into the development of OC and the identification of biomarkers targeting metabolic aspects. The focus of this review is to examine how abnormal lactate concentrations impact tumor immune evasion through various pathways involving immune cell differentiation, metabolism, and function. By uncovering targets associated with aerobic glycolysis and lactate metabolism in regulating tumor immunity, new opportunities for immunotherapy arise. A comprehensive understanding of how metabolites produced during aerobic glycolysis influence immune cell function has the potential to greatly enhance the effectiveness of immunotherapy treatments. Although there are currently challenges hindering the clinical application of combining immunotherapy with inhibitors targeting glycolysis or lactate metabolism, further extensive research in these areas is expected to facilitate their utilization in improved combination regimens for OC treatment.

## Data Availability

No datasets were generated or analysed during the current study.
